# Development and validation of interprofessional learning assessment tool for health professionals in continuing professional development (CPD)

**DOI:** 10.1371/journal.pone.0211405

**Published:** 2019-01-25

**Authors:** Irvin L. Ong, Michael Joseph S. Diño, Maria Minerva P. Calimag, Fe A. Hidalgo

**Affiliations:** 1 The Graduate School, University of Santo Tomas, Manila, Philippines; 2 Research Development and Innovation Center, Our Lady of Fatima University, Valenzuela City, Philippines; 3 Phi Gamma Chapter, Sigma Theta Tau International Honor Society in Nursing, Indianapolis, IN, United States of America; Warszawski Uniwersytet Medyczny, POLAND

## Abstract

**Introduction:**

Interprofessional learning (IPL) is increasingly recognized as a promising strategy in addressing the burgeoning challenges in healthcare. Its assessment remains to be perplexing and requires accurate measurements. Thus, this study intended to develop a valid and reliable reflective tool in assessing IPL as a Continuing Professional Development (CPD) outcome.

**Methods:**

A one-group post-posttest pre-experimental design with tool development was employed to establish the validity and reliability of the “Inventory of Reflective Vignette–Interprofessional Learning” (IRV-IPL). This tool was developed from an extensive literature review and designed with three segments to assess interprofessional competencies before, after, and what if scenarios using vignettes. After it was validated by education experts (*n* = 5) and written consent forms were signed by the participants, the IRV-IPL was pilot tested among healthcare professionals (*n* = 10) for analysis and improvement. During the actual implementation, it was administered to healthcare professionals (*n* = 45) who participated in a university-provided CPD event. Collected data underwent validity and reliability testing.

**Results:**

IRV-IPL generated excellent internal consistency (α = 0.98), and across all segments of collaboration (α = 0.96), coordination (α = 0.96), cooperation (α = 0.96), communication (α = 0.97), and commendation (α = 0.98). Items exhibited significantly positive large correlations (r > 0.35, p < 0.05) in all segments showing beneficial measures for postdictive validity in recalling prior interprofessional competencies, and predictive validity in estimating interprofessional learning as an outcome of CPD and alternative interventions.

**Conclusion:**

This study provided a piece of groundwork evidence on the use of IRV-IPL as a reflective assessment tool for interprofessional learning in CPD contexts. Further studies are essential to explore the educational utility of IRV framework in crafting relevant assessments and to establish construct validity of IRV-IPL using exploratory and confirmatory factor analyses.

## Introduction

The contemporary society becomes increasingly cognizant of collaboration’s role in achieving better health outcomes [1–8], as well as learning outcomes [9–14]. Inevitably, the global health community calls for the development of *interprofessional competencies* aside from the discipline-specific core competencies [1,7,8,15]. Even though several professional and specialty organizations may differ in their competency maps like the World Health Organization [8,16], Institute of Medicine [17,18], Interprofessional Education Collaborative [19], Alliance for Continuing Education in the Health Professions [15,20], and Consortium of Universities for Global Health [21,22], the prevailing emphases include safety, ethicality, professionalism, teamwork, collaboration and communication. Collaboration as an important construct, in particular, denotes a ‘higher level process that encompasses many frequently studied constructs such as, cooperation, teamwork, and coordination” [23]. These interprofessional competencies along with collaboration, reveal the urgency and necessity for *Interprofessional Education (IPE)* and *Learning (IPL)* to resolve the persisting professional silos towards team-based relations in the healthcare settings [3–5,8,24–26], and prevent practice-related errors and negative outcomes leading to compromised safety [27–29] of patients in the healthcare field. Notably, interprofessional learning mostly occurs in a CPD [30,31] or specifically *Continuing Interprofessional Education (CIPE)* [32] events.

As the interplay of collaboration, interprofessional competencies and learning become more valued, assessment in these areas are increasingly important [30]. Numerous studies display a variety of interprofessional assessments using different approaches and measures [33,34] For instance, Morison & Stewart [33] developed specific performance and program assessments. In both tools, the behavioral indicators include professional knowledge, performance, communication, and teamwork. Meanwhile, the readiness for interprofessional learning scale (RIPLS) consists of teamwork, identity, and roles constructs [35,36]. Curran & colleagues [37], for their part, developed an interprofessional collaborator assessment rubric (ICAR) with criteria on communication, collaboration, roles and responsibilities, collaborative client-centered approach, team functioning, and conflict resolution. Recently, Hayward and others [38] have revised this rubric, yet they still have the same categories. Similarly, the assessment of interprofessional team collaboration (AITCS) comprised of partnership, cooperation, and coordination subscales [39]. In assessing global curricular outcomes, Arif et al. [40] devised a survey tool with patient care and professionalism domains. For its part, the Agency for Health Research and Quality [41] released the team strategies and tools to enhance performance and patient safety (TeamSTEPPS), and developed tools for assessing teamwork and communication such as the Teamwork Perceptions Questionnaire (T-TPQ), Teamwork Attitudes Questionnaire (T-TAQ), Team Assessment Questionnaire (TAQ) and Team Performance Observation Tools (TPOT). These instruments have focused on team structure, leadership, situation monitoring, mutual support and communication domains [3,42,43]. Aside from T-TAQ, Brock et al. [3] utilized their AMUSE model in assessing interprofessional competence in terms of attitude, motivation, utility, and self-efficacy. Likewise, other interprofessional assessment combinations [44,45] may include attitudes toward health care teams scale (ATHCTS) with care quality and physician centrality subscales [46], interprofessional education perception scale (IEPS) with competency, cooperation needs, and actual cooperation subscales [47], and interprofessional collaboration (IPC) scale [48] with communication, accommodation and isolation factors. Whereas, other scholars developed their assessments for their own programs in general [2,49–56].

Interprofessional learning is seen beneficial in promoting quality, sustainable and safe healthcare practice [44]. Parsimoniously, many existing interprofessional assessments are mainly focused on measuring attitude and perceptions [57] that makes an assessment of interprofessional learning challenging [5,58]. Currently, there are limited attempts to reliably measure the impact of interprofessional learning constructs in healthcare education [59], and the demand for an assessment tool is becoming increasingly important [17,60]. Consequently, there is a lack of a reflective tool to measure the indirect effects of interprofessional interactions in many CPD, which in this paper refers to any lifelong learning activities among health professionals. Thus, this study aimed to fill this current gap through the development and psychometric assessment of a reflective tool that can be used for CPD involving IPL.

## Methods

### Research design

A *one-group posttest only* (also *one-shot case study* or *one-shot experiment*) with tool development design suits the existing contextual circumstances of the study. In this pre-experimental subdesign, the effect on the outcome is measured after a single group of participants has received a predetermined intervention [61,62]. This study specifically adapted the Ruzafa-Martinez et al. [63] tool development procedures to ensure the integrity of the collected data, which comprises of five (5) steps, namely: (a) *content design* mediated by review methods (e.g. literature review and existing tool synthesis), (b) *content validation* by field experts, (c) *pilot testing* with health professionals, (d) *preliminary analysis* for initial reliability and validity, and (e) *actual implementation* with final validation and analysis.

#### Content design

The content design of the *Inventory of Reflective Vignette (IRV)* as a framework for tool development considers the strategy of MacDonald, Stodel, Thompson, & Casimiro [64] and Bottenberg et al. [44] in embedding the pretest items into the posttest survey to minimize possible bias. MacDonald & colleagues [65] referred to this as the “*post-posttest design*,*”* which enables improvement in the tool sensitivity as well as reflection on prior and current conditions. To achieve these goals, the tool incorporates the use of research vignette. At large, a vignette question presents a carefully designed situation to a respondent [62]. Several studies [66,67] have combined vignette and questionnaire in measuring the variables and validating research instrument. In this case, the vignette on conventional CPD delivery (i.e. didactics or lectures) creates an opportunity to surface the construed judgments of the participants in connection with their previous (or vicarious) experiences.

The assessment of Interprofessional Learning (IRV-IPL) was constructed using the designed IRV framework. This innovative instrument is divided into two columns, one for the assessment items and another for the rating responses. A 6-point Likert-type scale (i.e., 1 = Emerging; 2 = Developing; 3 = Minimal; 4 = Proficient; 5 = Advanced; 6 = Excellent) is devised to allow deeper reflection, yet eliminate a neutral value for clearer measurement. Despite the advantage of having a midpoint as a respite for sensitive topics, this study involves mature health professionals, who are presumably more thoughtful and critical. The latter column is further divided into three segments for (a) before CPD event, (b) after CPD participation, and (c) a vignette (i.e. if participated in a traditional lecture format). Additionally, the instruments include a section for the respondent information.

#### Content validation

**Fig 1:**
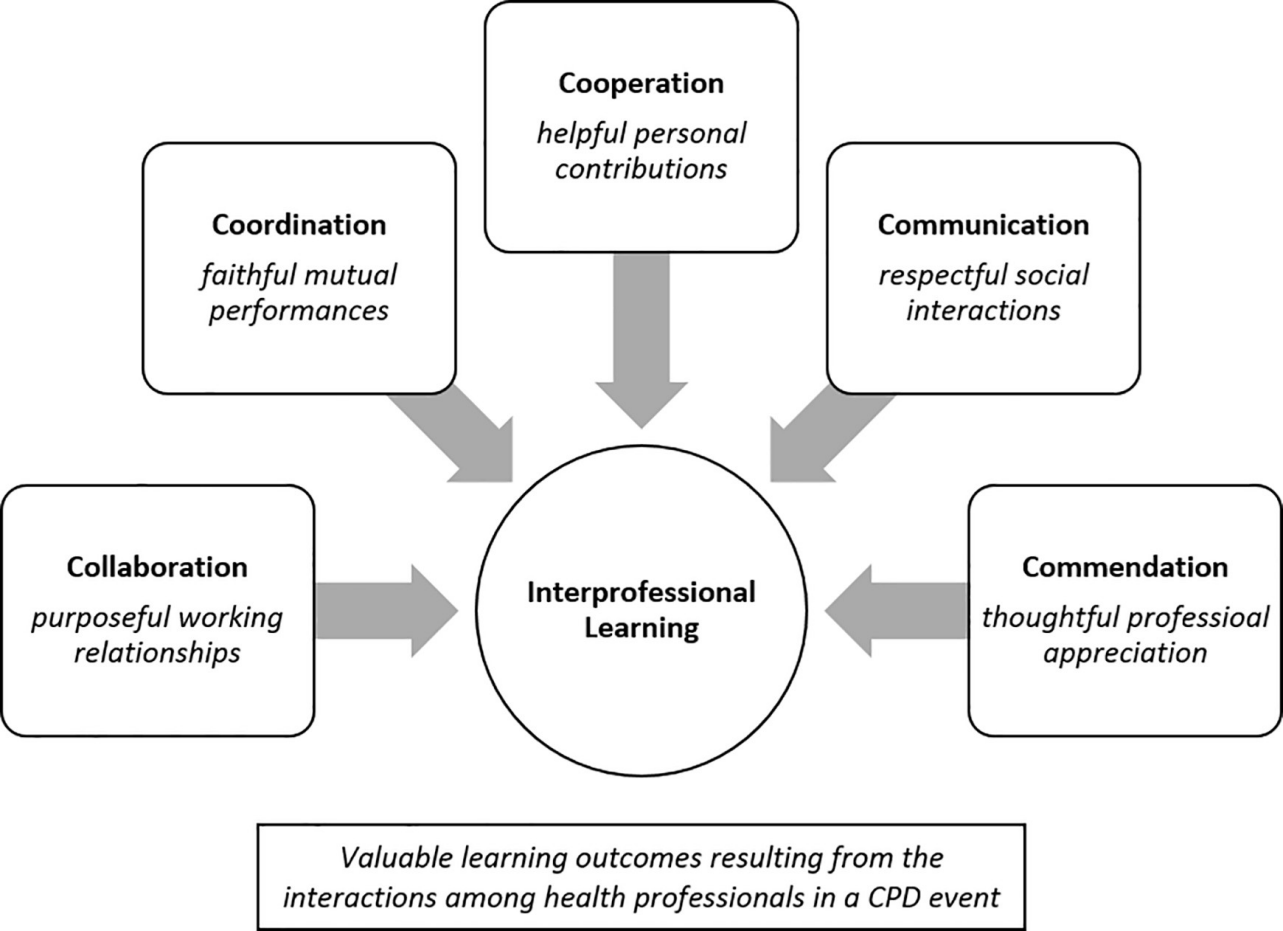
Identified IPL constructs as outcomes.

During the content validation, the study identified five IPL constructs (Fig 1) using synthesized evidences based on relevant literature review. There are five essential constructs for interprofessional learning in spite of the program purpose, namely: (a) collaboration, (b) coordination, (c) cooperation, (d) communication and (e) commendation. To begin with, collaboration centers on purposeful creation of a certain outcome. This coincides on the view that collaboration focuses on working relationships with others [37] in achieving a common goal [68]. Secondly, coordination seeks to inform other units in ensuring harmony leading towards a single direction. This explicitly emphasizes awareness of the action, but not so much on the results. As for cooperation, it highlights making contributions in a team [39]. Although this allows sharing thoughts and working together, it also fosters divergent thinking. Now, communication respectfully expresses information with others for understanding. This may include verbal and non-verbal strategies, as well as transmission and acquisition activities [33,37,48]. Lastly, commendation is conceptually described as the appreciation of others’ competencies, accomplishments, performances, professions, roles, and identities. These attributes may offer responsive if not pervasive (i.e. direct effect on health outcomes) measures of interprofessional learning.

The constructs of the *IRV-IPL* tool were reviewed independently by experts (*n* = 5) for relevance, applicability, and improvement.

#### Pilot testing and preliminary analysis

The designed tool was pilot tested on October 2017 in a university offering a CPD program for health professionals (*n* = 10). The institution was accredited by the National Professional Regulation Commission in delivering CPD programs.

### Data collection on the actual implementation

During the actual implementation on November 2017, the IRV-IPL was administered to health professionals (*n* = 45), who completed the university-provided CPD programs with constructivist teaching methods, which allow participants to learn interactively and to work primarily in groups. CPD units were given to the participants after successful completion. The participants in the pilot and actual testing were both registered and practicing physicians, nurses, and allied professionals like pharmacists, technicians, and therapists.

### Data analysis

The collected data was processed using *IBM SPSS Statistics version 22* to estimate its internal reliability using Cronbach’s alpha and validity coefficient using item-total correlation.

### Ethical approval

Our Lady of Fatima University Institutional Ethics Review Committee–Level 2 Accredited Research Ethics Committee of the Philippine Health Research Ethics Board approved this study. All participants (pilot and actual) signed a written informed consent after careful orientation on the study objectives and plan.

## Results

The study analyzed the utilization of the IRV-IPL in assessing interprofessional learning among health professionals during the pilot (n = 10) and actual (n = 45) implementations.

### Preliminary analysis: Pilot test reliability and validity

The initial test reliability analysis (Table 1) showed that IRV-IPL is a highly reliable instrument (α = 0.97) with excellent internal consistency in each segment that is before (α = 0.96), after (α = 0.95), and if (α = 0.99). In terms of the identified construct, collaboration (α = 0.87) and communication (α = 0.82) had good internal reliability, while coordination (α = 0.94), cooperation (α = 0.92) and commendation (α = 0.97) showed excellent measures.

**Table 1:** Pilot test reliability (*n* = 10).

Constructs (n of items)	Before	After	If	Overall
Collaboration (*n* = 5)	0.80	0.85	0.98	0.87
Coordination (*n* = 5)	0.95	0.88	0.99	0.94
Cooperation (*n* = 5)	0.95	0.83	0.99	0.92
Communication (*n* = 5)	0.74	0.83	0.90	0.82
Commendation (*n* = 5)	0.96	0.96	0.98	0.97
Overall	0.96	0.95	0.99	0.97

The pilot study also tested IRV-IPL for statistical validity (Table 2) by correlating each item with the total score (i.e. the sum of all segments). Given that the tool underwent content and face validation, it can serve as a valid measure of interprofessional learning. The validity coefficients showed the inconsistency of estimates resulting in items with insignificant correlations (*p* > 0.05) with exception to the construct of commendation. Based on the initial validation and focus group discussion, most items were reviewed and reworded to improve further its measures.

**Table 2:** Pilot validity testing (*n* = 10).

IPL Constructs and Items	Segments (r)		
	Before	After	If
Collaboration			
*Work well with the team members*	0.77*	0.75*	0.90*
*Seek others to work together*	0.73*	0.49	0.91*
*Include other team members*	0.18	0.36	0.90*
*Use a team approach*	0.50	0.58	0.89*
*Explain the roles/tasks*	0.55	0.44	0.91*
Coordination			
*Negotiate tasks/responsibilities with others*	0.75*	0.70*	0.91*
*Inform other participants for any changes*	0.63*	0.33	0.91*
*Work well with other groups*	0.66*	0.39	0.96*
*Discuss with others*	0.70*	0.47	0.92*
*Know the work of others*	0.61	0.70*	0.95*
Cooperation			
*Share my abilities with others*	0.66*	0.61	0.84*
*Be responsible to the team*	0.52	0.43	0.81*
*Show my support/concern*	0.58	0.49	0.87*
*Offer useful information*	0.44	0.09	0.81*
*Help other participants*	0.59	0.25	0.80*
Communication			
*Listen to others*	-0.10	0.28	0.40
*Express my concerns*	0.07	0.44	0.75*
*Encourage others to ask*	0.56	0.45	0.78*
*Share my thoughts*	-0.09	0.26	0.60
*Manage conflict*	-0.10	0.44	0.74*
Commendation			
*Give constructive feedbacks to others*	0.81*	0.87*	0.67*
*Show trust in others while learning/working*	0.80*	0.73*	0.79*
*Recognize the performance of others*	0.75*	0.62*	0.69*
*Appreciate the contributions of others*	0.91*	0.65*	0.77*
*Consider the inputs/ideas of others*	0.89*	0.67*	0.67*

*Significant at 0.05 alpha level

### Actual implementation: Descriptive test responses to IRV-IPL

As shown (Table 3), the highest mean responses of the participants on the IPL items are consistently observed after the CPD program. All the ratings fall within the range of 4.5–5.49 or Proficient.

**Table 3:** IRV-IPL test responses (*n* = 45).

Interprofessionalism in Learning	Before		After		If	
	Mean	SD	Mean	SD	Mean	SD
Collaboration						
*Work well with the team members*	3.44	1.24	4.80	0.99	3.60	1.14
*Seek others to work together*	3.38	1.15	4.82	0.98	3.60	1.03
*Include other team members*	3.44	1.27	5.02	0.89	3.59	1.04
*Use a team approach*	3.67	1.21	5.04	1.00	3.69	1.14
*Explain the roles/tasks*	**3.37**	1.11	4.51	1.42	**3.33**	1.28
Coordination						
*Negotiate tasks/responsibilities with others*	3.36	1.09	4.62	0.91	3.42	1.36
*Inform other participants for any changes*	3.36	1.09	4.80	0.89	3.64	1.12
*Work well with other groups*	3.42	1.12	4.78	0.97	3.64	1.22
*Discuss with others*	3.44	1.18	4.71	0.90	3.64	1.20
*Know the work of others*	3.47	1.12	4.73	0.91	3.61	1.22
Cooperation						
*Share my abilities with others*	3.49	1.18	4.98	1.03	3.89	1.10
*Be responsible to the team*	3.76	1.11	5.11	0.91	3.91	1.07
*Show my support/concern*	3.67	1.04	5.09	0.87	3.96	1.06
*Offer useful information*	3.60	1.07	4.96	1.02	3.80	1.02
*Help other participants*	3.84	1.13	5.00	0.95	3.93	1.07
Communication						
*Listen to others*	3.82	1.03	5.11	0.88	4.04	1.17
*Express my concerns*	3.91	1.18	5.04	0.98	3.96	1.19
*Encourage others to ask*	3.71	1.18	4.98	0.97	3.96	1.11
*Share my thoughts*	3.82	1.01	5.00	0.95	3.89	1.13
*Manage conflict*	3.82	0.98	5.02	0.87	3.91	1.16
Commendation						
*Give constructive feedbacks to others*	3.44	1.08	4.76	0.93	3.80	1.12
*Show trust in others while learning/working*	3.67	1.13	5.00	0.98	3.84	1.17
*Recognize the performance of others*	3.80	1.20	5.04	1.00	3.82	1.27
*Appreciate the contributions of others*	3.80	1.27	5.07	1.01	3.89	1.27
*Consider the inputs/ideas of others*	3.80	1.20	5.09	1.00	3.93	1.25

Note: 1.0–1.49 Emerging; 1.5–2.49 Developing; 2.5–3.49 Minimal; 3.5–4.49 Advanced; 4.5–5.49 Proficient 5.5–6.0 Excellent

Ratings on the instrument “*if lecture”* was used as an intervention are consistently higher compared to the ratings before the CPD program. Most of the items in “if lecture was used” fall within the range of 3.5–4.49 or Advanced. Two items namely (1) *Explain the roles/tasks of each team member* and (2) *Negotiate tasks/responsibilities with other participants* which fall within the rage of 2.5–3.49 of Minimal.

Evaluations before CPD fall within two ranges 2.5–3.49 (Minimal) and 3.5–4.49 (Advanced). Ratings on the Coordination dimension fall within the range of 2.5–3.49 Minimal while the ratings on Communication dimension fall within 3.5–4.49 or Advanced. Majority of the items under Commendation fall within 3.5–4.49 or Advanced. The only item “*Give constructive feedbacks to other participants*” fall within 2.5–3.49 or Minimal. Majority of the items under Collaboration fall within 2.5–3.49 or Minimal. Only item “*Use team an approach to achieve the goals/outcomes*” fall within 3.5–4.49 or Advanced.

### Actual implementation: Reliability and validity

The designed tool demonstrated excellent internal consistency (α = 0.98) across all segments (Table 4). As compared to the pilot test, there was also notable increased reliability estimates among the identified constructs, namely: collaboration (α = 0.96), coordination (α = 0.96), cooperation (α = 0.96), communication (α = 0.97), and commendation (α = 0.98). This suggests that IRV-IPL was able to measure consistently the identified constructs, which in turn could provide a reliable measure of IPL.

**Table 4:** IRV-IPL test reliability (*n* = 45).

Constructs (n of items)	Before	After	If	Overall
Collaboration (*n* = 5)	0.93	0.96	0.98	0.96
Coordination (*n* = 5)	0.95	0.95	0.99	0.96
Cooperation (*n* = 5)	0.95	0.96	0.98	0.96
Communication (*n* = 5)	0.96	0.97	0.98	0.97
Commendation (*n* = 5)	0.98	0.97	0.99	0.98
Overall	0.98	0.98	0.99	0.98

Further analysis (Table 5) shows significant positive limited relationship (*r* < 0.70, *p* < 0.05) across all IPL constructs when (a) before and after, and (b) before and if were correlated. Moreover, this is specifically present when correlating after and if segments in the construct of coordination. This perhaps may imply the distinction of each segment. It means that IRV-IPL can also provide reliable and reflective assessments of (a) baseline interprofessional competencies, (b) IPL as outcomes of an intervention, and (c) comparative IPL measure for alternative situations.

**Table 5:** Relation matrix of IPL constructs as to segments.

Constructs	Segments (r)		
	Before	After	If
Collaboration			
Before	-		
After	0.45*	-	
If	0.50*	0.22	-
Coordination			
Before	-		
After	0.49*	-	
If	0.55*	0.33*	-
Cooperation			
Before	-		
After	0.31*	-	
If	0.44*	0.09	-
Communication			
Before	-		
After	0.30*	-	
If	0.63*	0.11	-
Commendation			
Before	-		
After	0.48*	-	
If	0.52*	0.23	-

*Significant at 0.05 alpha level

The actual validity coefficients (Table 6) of the corresponding items for all constructs exhibited significantly positive large correlations (*r* > 0.35, *p* < 0.05) in all segments except for one item (i.e. cooperation before—*Show my support/concern for other participants*). Such a remarkable result can be attributed to the effective strategy used in improving tool items during pilot testing. This clearly indicates the established validity of IRV-IPL as an assessment tool. In addition, the instrument was able to measure interprofessional learning at different segments. It also shows that IRV-IPL demonstrated beneficial measures for postdictive validity in recalling prior interprofessional competencies, and predictive validity in estimating IPL as an outcome of CPD and alternative interventions.

**Table 6:** Item-total validity measures of IRV-IPL.

IPL Constructs and Items	Segments (r)		
	Before	After	If
Collaboration			
*Work well with the team members*	0.72*	0.72*	0.74*
*Seek other members to accomplish the work*	0.77*	0.62*	0.70*
*Include other team members in making plans/decisions*	0.67*	0.61*	0.68*
*Use a team approach to achieve the goals/outcomes*	0.75*	0.56*	0.66*
*Explain the roles/tasks of each team member*	0.77*	0.71*	0.74*
Coordination			
*Negotiate tasks/responsibilities with other participants*	0.77*	0.60*	0.79*
*Inform other participants for any updates and changes*	0.65*	0.65*	0.82*
*Work well with the participants of other groups*	0.79*	0.70*	0.80*
*Discuss your plans/actions with other participants*	0.79*	0.61*	0.82*
*Know the work/responsibility of other participants*	0.78*	0.74*	0.77*
Cooperation			
*Share my inputs/abilities with other participants*	0.75*	0.54*	0.74*
*Be responsible with my contributions to the team*	0.65*	0.55*	0.68*
*Show my support/concern for other participants*	-0.09	0.47*	0.70*
*Offer useful information to other participants*	0.77*	0.56*	0.74*
*Help other participants when necessary*	0.74*	0.56*	0.67*
Communication			
*Listen actively to other participants*	0.82*	0.61*	0.77*
*Express my concerns in a professional manner*	0.79*	0.58*	0.82*
*Encourage others to ask useful questions politely*	0.77*	0.51*	0.79*
*Share my thoughts in a clear effective manner*	0.83*	0.49*	0.79*
*Manage conflict in a courteous manner*	0.85*	0.50*	0.72*
Commendation			
*Give constructive feedbacks to other participants*	0.82*	0.64*	0.73*
*Show trust in other participants while learning/working*	0.85*	0.63*	0.77*
*Recognize the performance of other participants*	0.82*	0.65*	0.76*
*Appreciate the contributions of other participants*	0.82*	0.68*	0.77*
*Consider the inputs/ideas of other participants*	0.82*	0.66*	0.76*

*Significant at 0.05 alpha level

## Discussion

### IRV-IPL as a reliable assessment tool

The IRV-IPL exhibited good psychometric properties as evidenced by a remarkably high internal consistency coefficient score. This tool possesses inherent characteristics of a reliable instrument–suitability of length, validity of content, practicality of administration, inclusivity of user, usability of tool, and clarity of structure.

The IRV-IPL’s accuracy to measure interprofessional learning is beneficial in generating consistently reproducible results under related conditions and subjects [69,70] on several items under the areas of collaboration, coordination, cooperation, communication, and commendation. Measurement of these constructs is significant for it measures collaboration attribute as a central concept of interprofessional learning. For instance, a healthcare professional with the inability to work together with other members of the healthcare team may translate to poor team learning outcomes and eventually to poor healthcare delivery in practice [71,72]. In evidence, most of the tools developed to assess interprofessional learning focuses on the quality of interpersonal interactions and behavior within teams [59,73]. As an outcome of positive interactions, healthcare workers who are members of interprofessional groups are expected to function cohesively [74] under shared leadership, decision-making and co-shared accountability [75].

### IRV-IPL as a valid assessment tool

IRV-IPL is a valid measurement of interprofessional learning through its constructs that were carefully crafted using global evidences found in previous studies and enhanced through effective negotiations between tool designers and users. It is composed of harmonized relevant key elements of interprofessional learning broken down into core values (i.e. Collaboration, Coordination, Cooperation, Communication, and Commendation). The tool clearly distinguishes one construct to another through concrete definitions. This is substantial since interprofessional learning involves a variety of attributes of interprofessionalism [76] beyond generic and profession-specific competencies [77]. Previous studies (e.g. [78]) underscored the need to identify individual features which is essential in identifying areas of strength and weakness in learning.

The validity of the IRV-IPL as a tool is useful to educators who intend to come up with an assessment backed by evidence and theoretical supports [79] to interpret interprofessional learning regardless of the type of healthcare provider test takers [80,81]. By and large, learning measurement under interprofessional scope is incommensurable even under same learning outcomes for different purposes. Interprofessional learning as a form of educational exercise was acknowledged to occur in divergent environments due to differing professional standards, social structures, individual responses, learner behaviors, and learning resources [82]. Education planners may benefit from valid assessment tools as sources of evidence-based data for the curriculum and instructional development and improvement from profession-specific to interprofession-sensitive learning. Interestingly, IRV-IPL focuses on positive affinity toward IPL in contrast with other tools that include both exemplar and non-exemplar performances. This may lead to an acceptable conclusion based on sound reasoning, evidence, and justification, and further enhance tool sensitivity [83].

### IRV-IPL as a reflective assessment tool

IRV-IPL is a tool that promotes reflection through critical introspection. It involves the use of an odd-response scale that removes neutrality, promotes thoughtfulness, compels looking back, and minimizes midpoint bias. Following a post-posttest design that integrates pretest and posttest in a single administration preventing tool desensitization, it can be administered to economically obtain viable comparisons between self-assessments in interprofessional learning and alternative environments.

When juxtaposed to previous tools, an apparent highlight of the IRV-IPL is the advantage of evaluating educational impact through reflective assessments and parallel evaluation of current and previous understandings through the innovative use of anchoring vignettes [84,85]. This approach fosters deeper contemplation of past experiences in foreseeing possible outcomes given a different context. In this study, anchoring vignettes pertain to the hypothetical case [86] of the respondents in a lecture scenario which can be rooted from their previous experiences. Vignettes that underscore “historical moments” [87] are proven effective in comparing insights in various life courses occurring in different levels [88] and are positively correlated with actual outcomes [89].

## Conclusion

The study offered a valid and reliable assessment tool for interprofessional learning (IPL) utilizing the Inventory of Reflective Vignette (IRV) as a framework for tool development. Based on the study findings, IRV-IPL can measure IPL consistently due to its excellent internal reliability and capture variances among segments using post-posttest strategy. It also demonstrated face and content validity supported by significant beneficial item-total correlations. Although the study was limited by the number and demographics of samples, it was able to provide empirical evidences to substantiate IRV-IPL as a reflective tool. Health professions leaders and educators are encouraged to use this tool in assessing IPL so as to identify appropriate policies, strategies, and interventions. Further studies are needed to: (a) explore the educational utility of IRV framework in designing relevant assessments, (b) investigate demographic influence on IRV-IPL by finding its determinants and correlates, (c) strengthen statistical evidences for IRV-IPL’s criterion validity by identifying appropriate criteria, and (d) establish construct validity of IRV-IPL using exploratory and confirmatory factor analyses. It is hoped that the IRV-IPL can provide a better IPL estimate to promote interprofessionalism in achieving better healthcare outcomes through collaboration.

## Supporting information

S1 Tool Inventory of reflective vignettes–interprofessional learning (IRV-IPL). (PDF)

## References

[1] Balmer JT. The transformation of continuing medical education (CME) in the United States. Adv Med Educ Pract. 2013; 171 10.2147/AMEP.S35087 PMC3791543 24101887

[2] Braun HJ, O’Sullivan PS, Dusch MN, Antrum S, Ascher NL. Improving interprofessional collaboration: Evaluation of implicit attitudes in the surgeon–nurse relationship. Int J Surg. 2015;13: 175–179. 10.1016/j.ijsu.2014.11.032 25497005

[3] Brock D, Abu-Rish E, Chiu C-R, Hammer D, Wilson S, Vorvick L, et al Interprofessional education in team communication: working together to improve patient safety. BMJ Qual Saf. 2013;22: 414–423. 10.1136/bmjqs-2012-000952 23293118

[4] Failla KR, Macauley K. Interprofessional Simulation: A Concept Analysis. Clin Simul Nurs. 2014;10: 574–580. 10.1016/j.ecns.2014.07.006

[5] Hays R. Interprofessional education. Clin Teach. 2013;10: 339–341. 10.1111/tct.12115 24015744

[6] Sierpina VS, Kreitzer MJ. Interprofessional Education and Integrative Healthcare. EXPLORE J Sci Heal. 2014;10: 265–266. 10.1016/j.explore.2014.04.011 25037672

[7] Wilson L, Callender B, Hall TL, Jogerst K, Torres H, Velji A. Identifying Global Health Competencies to Prepare 21st Century Global Health Professionals: Report from the Global Health Competency Subcommittee of the Consortium of Universities for Global Health. J Law Med Ethics. 2014;42: 26–31. 10.1111/jlme.12184 25564707

[8] World Health Organization. Transforming and Scaling Up Health Professionals’ Education and Training: World Health Organization Guidelines 2013 [Internet]. Geneva, Switzerland: World Health Organization; 2013. Available: http://www.who.int/hrh/resources/transf_scaling_hpet/en/26042324

[9] Bolderston A. Maintaining competence: a holistic view of continuous professional development. J Radiother Pract. 2007;6 10.1017/S1460396907005031

[10] Chipchase LS, Johnston V, Long PD. Continuing professional development: The missing link. Man Ther. 2012;17: 89–91. 10.1016/j.math.2011.09.004 22018439

[11] Cruz-Correia R. AprendIS: A Tool for (in)Formal Learning in Health Informatics. Procedia Technol. 2014;16: 1367–1373. 10.1016/j.protcy.2014.10.154

[12] Kumar S. Signature pedagogy, implementation and evaluation of an online program that impacts educational practice. Internet High Educ. 2014;21: 60–67. 10.1016/j.iheduc.2013.11.001

[13] Lawson C, Cowling C. Social media: The next frontier for professional development in radiography. Radiography. 2015;21: e74–e80. 10.1016/j.radi.2014.11.006

[14] Shaidullin RN, Safiullin LN, Gafurov IR, Safiullin NZ. Blended Learning: Leading Modern Educational Technologies. Procedia—Soc Behav Sci. 2014;131: 105–110. 10.1016/j.sbspro.2014.04.087

[15] Balmer JT. The Alliance for Continuing Education in the Health Professions: A Brief Overview of Health Care CE Professionals. Dickerson PS, Lubejko BG, editors. J Contin Educ Nurs. 2014;45: 153–154. 10.3928/00220124-20140327-11 24702049

[16] World Health Organization. Sexual and reproductive health core competencies in primary care: attitudes, knowledge, ethics, human rights, leadership, management, teamwork, community work, education, counselling, clinical settings, service, provision. Santé sexuelle et reproductive compétences de base en soins primaires. 2011; Available: http://apps.who.int/iris/handle/10665/44507

[17] Committee on Measuring the Impact of Interprofessional Education on Collaborative Practice and Patient Outcomes, Board on Global Health, Institute of Medicine. Measuring the Impact of Interprofessional Education on Collaborative Practice and Patient Outcomes [Internet]. Washington, D.C.: National Academies Press; 2015 10.17226/21726 26803876

[18] National Research Council (U.S.), Institute of Medicine (U.S.), editors. Health professions education: a bridge to quality. Washington, D.C.; Oxford: National Academies; Oxford Publicity Partnership; 2004.

[19] Resources. In: IPEC Interprofessional Education Collaborative [Internet]. [cited 30 Apr 2018]. Available: http://ipecollaborative.org/resources.html

[20] Alliance for Continuing Education in the Health Professions: National Learning Competencies [Internet]. [cited 30 Apr 2018]. Available: http://www.acehp.org/p/cm/ld/fid=15

[21] Proposed Interprofessional Global Health Competencies | Consortium of Universities for Global Health [Internet]. [cited 30 Apr 2018]. Available: https://www.cugh.org/forums/teaching-global-health-competenciescurricula-methods-and-evaluation

[22] Jogerst K, Callender B, Adams V, Evert J, Fields E, Hall T, et al Identifying Interprofessional Global Health Competencies for 21st-Century Health Professionals. Ann Glob Health. 2015;81: 239–247. 10.1016/j.aogh.2015.03.006 26088089

[23] Bedwell WL, Wildman JL, DiazGranados D, Salazar M, Kramer WS, Salas E. Collaboration at work: An integrative multilevel conceptualization. Hum Resour Manag Rev. 2012;22: 128–145. 10.1016/j.hrmr.2011.11.007

[24] Aase I, Aase K, Dieckmann P. Teaching interprofessional teamwork in medical and nursing education in Norway: A content analysis. J Interprof Care. 2013;27: 238–245. 10.3109/13561820.2012.745489 23205762

[25] Becker KL, Hanyok LA, Walton-Moss B. The Turf and Baggage of Nursing and Medicine: Moving Forward to Achieve Success in Interprofessional Education. J Nurse Pract. 2014;10: 240–244. 10.1016/j.nurpra.2014.02.004

[26] Watts P, Langston SB, Brown M, Prince C, Belle A, Skipper MW, et al Interprofessional Education: A Multi-patient, Team-Based Intensive Care Unit Simulation. Clin Simul Nurs. 2014;10: 521–528. 10.1016/j.ecns.2014.05.004

[27] Axley L. Competency: A Concept Analysis. Nurs Forum (Auckl). 2008;43: 214–222. 10.1111/j.1744-6198.2008.00115.x 19076465

[28] Riley BA, Riley G. Innovation in graduate medical education–using a competency based medical education curriculum. Int J Osteopath Med. 2017;23: 36–41. 10.1016/j.ijosm.2016.07.001

[29] Mann JE, Amerine LB, Waldron K, Wolcott MD, McLaughlin JE. Pharmacist perceptions of competency: Identifying priority areas for a competency program development at an academic medical center. Res Soc Adm Pharm. 2017; 10.1016/j.sapharm.2017.07.008 28754424

[30] Barr H, Low H. Introducing Interprofessional Education [Internet]. United Kingdom: CAIPE; 2013 Available: http://caipe.org.uk/silo/files/introducing-interprofessional-education.pdf

[31] Stone J. Moving interprofessional learning forward through formal assessment. Med Educ. 2010;44: 396–403. 10.1111/j.1365-2923.2009.03607.x 20444075

[32] Simmons B, Wagner S. Assessment of continuing interprofessional education: Lessons learned. J Contin Educ Health Prof. 2009;29: 168–171. 10.1002/chp.20031 19728381

[33] Morison SL, Stewart MC. Developing interprofessional assessment. Learn Health Soc Care. 2005;4: 192–202. 10.1111/j.1473-6861.2005.00103.x

[34] Simmons B, Egan-Lee E, Wagner SJ, Esdaile M, Baker L, Reeves S. Assessment of interprofessional learning: the design of an interprofessional objective structured clinical examination (iOSCE) approach. J Interprof Care. 2011;25: 73–74. 10.3109/13561820.2010.483746 20645683

[35] Hood K, Cant R, Baulch J, Gilbee A, Leech M, Anderson A, et al Prior experience of interprofessional learning enhances undergraduate nursing and healthcare students’ professional identity and attitudes to teamwork. Nurse Educ Pract. 2014;14: 117–122. 10.1016/j.nepr.2013.07.013 23937910

[36] McFadyen AK, Webster V, Strachan K, Figgins E, Brown H, Mckechnie J. The Readiness for interprofessional learning scale: A possible more stable sub-scale model for the original version of RIPLS. J Interprof Care. 2005;19: 595–603. 10.1080/13561820500430157 16373215

[37] Curran V, Hollett A, Casimiro LM, Mccarthy P, Banfield V, Hall P, et al Development and validation of the interprofessional collaborator assessment rubric ((ICAR)). J Interprof Care. 2011;25: 339–344. 10.3109/13561820.2011.589542 21732723

[38] Hayward MF, Curran V, Curtis B, Schulz H, Murphy S. Reliability of the Interprofessional Collaborator Assessment Rubric (ICAR) in Multi Source Feedback (MSF) with post-graduate medical residents. BMC Med Educ. 2014;14 10.1186/s12909-014-0279-9 25551678 PMC4318203

[39] Orchard CA, King GA, Khalili H, Bezzina MB. Assessment of Interprofessional Team Collaboration Scale (AITCS): Development and testing of the instrument. J Contin Educ Health Prof. 2012;32: 58–67. 10.1002/chp.21123 22447712

[40] Arif SA, Dilich A, Ramel C, Strong S. Impact of an interprofessional international experience abroad on the attitudes of health care professional students. Curr Pharm Teach Learn. 2014;6: 639–645. 10.1016/j.cptl.2014.05.010

[41] Agency for Health Research and Quality. National Implementation Plan. In: TeamSTEPPS [Internet]. 11 2013 [cited 23 Apr 2015]. Available: http://teamstepps.ahrq.gov/aboutnationalIP.htm

[42] Center for Health Sciences Interprofessional Education, Research and Practice. Tools for Evaluation | CHSIERP. In: CHSIERP [Internet]. 2015 [cited 23 Apr 2015]. Available: http://www.collaborate.uw.edu/tools-and-curricula/tools-for-evaluation.html

[43] King PK, Szczerba FM, Rega PP, Peeters MJ. Simulation-based interprofessional education: Are we hitting the mark? Curr Pharm Teach Learn. 2014;6: 558–561. 10.1016/j.cptl.2014.03.004

[44] Bottenberg MM, DeWitt JE, Wall GC, Fornoff A, Stelter N, Soltis D, et al Assessment of interprofessional perceptions and attitudes of health professional students in a simulation laboratory setting. Curr Pharm Teach Learn. 2013;5: 167–174. 10.1016/j.cptl.2012.12.004

[45] Garrido M (Maite), Dlugasch L, Graber PM. Integration of Interprofessional Education and Culture into Advanced Practice Simulations. Clin Simul Nurs. 2014;10: 461–469. 10.1016/j.ecns.2014.06.001

[46] Heinemann GD, Schmitt MH, Farrell MP, Brallier SA. Development of an Attitudes toward Health Care Teams Scale. Eval Health Prof. 1999;22: 123–142. 10.1177/01632789922034202 10350960

[47] McFadyen AK, Maclaren WM, Webster VS. The Interdisciplinary Education Perception Scale (IEPS): An alternative remodelled sub-scale structure and its reliability. J Interprof Care. 2007;21: 433–443. 10.1080/13561820701352531 17654160

[48] Kenaszchuk C, Reeves S, Nicholas D, Zwarenstein M. Validity and reliability of a multiple-group measurement scale for interprofessional collaboration. BMC Health Serv Res. 2010;10: 83 10.1186/1472-6963-10-83 20353577 PMC2867963

[49] Busen NH. An Interprofessional Education Project to Address the Health Care Needs of Women Transitioning From Prison to Community Reentry. J Prof Nurs. 2014;30: 357–366. 10.1016/j.profnurs.2014.01.002 25150422

[50] Davis LI, Wright DJ, Gutierrez MS, Nam JJ, Nguyen J, Waite AT. Interprofessional global service learning: A pharmacy and nursing practice experience in Botswana. Curr Pharm Teach Learn. 2015;7: 169–178. 10.1016/j.cptl.2014.11.017

[51] Lagan C, Wehbe-Janek H, Waldo K, Fox A, Jo C, Rahm M. Evaluation of an Interprofessional Clinician–Patient Communication Workshop Utilizing Standardized Patient Methodology. J Surg Educ. 2013;70: 95–103. 10.1016/j.jsurg.2012.06.018 23337677

[52] Mann K, Sargeant J, Hill T. Knowledge translation in interprofessional education: what difference does interprofessional education make to practice? Learn Health Soc Care. 2009;8: 154–164. 10.1111/j.1473-6861.2008.00207.x

[53] Pfaff MA. Learning together: The image gently interprofessional simulation for nursing and allied health students. Teach Learn Nurs. 2014;9: 108–114. 10.1016/j.teln.2014.02.001

[54] Spoelstra H, Stoyanov S, Burgoyne L, Bennett D, Sweeney C, Drachsler H, et al Convergence and translation: attitudes to inter-professional learning and teaching of creative problem-solving among medical and engineering students and staff. BMC Med Educ. 2014;14: 14 10.1186/1472-6920-14-14 24450310 PMC3996181

[55] Steel A, Wardle J, Diezel H, Johnstone K, Adams J. Educating for collaboration: The outcomes of an interprofessional education workshop for complementary and alternative maternity care providers. Adv Integr Med. 2014;1: 17–24. 10.1016/j.aimed.2013.05.001

[56] Voest M de, Raguckas S, Bambini D, Beel-Bates C. Interprofessional teaching: An inter-university experience involving pharmacy and nursing students. Curr Pharm Teach Learn. 2013;5: 450–457. 10.1016/j.cptl.2013.06.004

[57] Lapkin S, Levett-Jones T, Gilligan C. A systematic review of the effectiveness of interprofessional education in health professional programs. Nurse Educ Today. 2013;33: 90–102. 10.1016/j.nedt.2011.11.006 22196075

[58] Gilbert JHV. Interprofessional–education, learning, practice and care. J Interprof Care. 2013;27: 283–285. 10.3109/13561820.2012.755807 23391027

[59] Wong AKC, Wong FKY, Chan LK, Chan N, Ganotice FA, Ho J. The effect of interprofessional team-based learning among nursing students: A quasi-experimental study. Nurse Educ Today. 2017;53: 13–18. 10.1016/j.nedt.2017.03.004 28340482

[60] Packard K, Ryan-Haddad A, Monaghan MS, Doll J, Qi Y. Application of validated instruments to assess university-wide interprofessional service-learning experiences. J Interprofessional Educ Pract. 2016;4: 69–75. 10.1016/j.xjep.2016.06.005

[61] DePoy E, Gitlin LN. Introduction to research: understanding and applying multiple strategies. Fifth edition St. Louis, Missouri: Elsevier; 2015.

[62] Lavrakas PJ, editor. Encyclopedia of survey research methods. Thousand Oaks, Calif: SAGE Publications; 2008.

[63] Ruzafa-Martinez M, Lopez-Iborra L, Moreno-Casbas T, Madrigal-Torres M. Development and validation of the competence in evidence based practice questionnaire (EBP-COQ) among nursing students. BMC Med Educ. 2013;13: 19 10.1186/1472-6920-13-19 23391040 PMC3598337

[64] MacDonald CJ, Stodel EJ, Thompson TL, Casimiro L. W(e)Learn: a framework for online interprofessional education. Int J Electron Healthc. 2009;5: 33–47. 10.1504/IJEH.2009.026271 19505867

[65] MacDonald CJ, Archibald D, Trumpower D, Casimiro L, Cragg B, Jelley W. Designing and Operationalizing a Toolkit of Bilingual Interprofessional Education Assessment Instruments. J Res Interprofessional Pract Educ Vol 1 No 3 2010 2010; Available: http://www.jripe.org/index.php/journal/article/view/36/35

[66] Agoritsas T, Deom M, Perneger TV. Study design attributes influenced patients’ willingness to participate in clinical research: a randomized vignette-based study. J Clin Epidemiol. 2011;64: 107–115. 10.1016/j.jclinepi.2010.02.007 20558036

[67] Das J, Hammer J. Which doctor? Combining vignettes and item response to measure clinical competence. J Dev Econ. 2005;78: 348–383. 10.1016/j.jdeveco.2004.11.004

[68] Oregon State Department of Education. Cooperation, Coordination and Collaboration: A Guide for Child Care and Head Start Programs. [Internet]. 1995 Available: http://search.ebscohost.com/login.aspx?direct=true&db=eric&AN=ED393558&site=ehost-live

[69] Nelson M. 8. The validation of dietary assessment. In: Margetts BM, Nelson M, editors. Design Concepts in Nutritional Epidemiology. Oxford University Press; 1997 pp. 241–272. 10.1093/acprof:oso/9780192627391.003.0008

[70] Ursachi G, Horodnic IA, Zait A. How Reliable are Measurement Scales? External Factors with Indirect Influence on Reliability Estimators. Procedia Econ Finance. 2015;20: 679–686. 10.1016/S2212-5671(15)00123-9

[71] Coleman MT, McLean A, Williams L, Hasan K. Improvement in interprofessional student learning and patient outcomes. J Interprofessional Educ Pract. 2017;8: 28–33. 10.1016/j.xjep.2017.05.003

[72] Granheim BM, Shaw JM, Mansah M. The use of interprofessional learning and simulation in undergraduate nursing programs to address interprofessional communication and collaboration: An integrative review of the literature. Nurse Educ Today. 2018;62: 118–127. 10.1016/j.nedt.2017.12.021 29331902

[73] Shrader S, Farland MZ, Danielson J, Sicat B, Umland EM. A Systematic Review of Assessment Tools Measuring Interprofessional Education Outcomes Relevant to Pharmacy Education. Am J Pharm Educ. 2017;81: 119 10.5688/ajpe816119 28970620 PMC5607729

[74] Derbyshire JA, Machin AI, Crozier S. Facilitating classroom based interprofessional learning: A grounded theory study of university educators’ perceptions of their role adequacy as facilitators. Nurse Educ Today. 2015;35: 50–56. 10.1016/j.nedt.2014.05.001 24933402

[75] Ketcherside M, Rhodes D, Powelson S, Cox C, Parker J. Translating interprofessional theory to interprofessional practice. J Prof Nurs. 2017;33: 370–377. 10.1016/j.profnurs.2017.03.002 28931485

[76] Hylin U, Lonka K, Ponzer S. Students’ approaches to learning in clinical interprofessional context. Med Teach. 2011;33: e204–e210. 10.3109/0142159X.2011.557410 21456979

[77] McAllister S, Lincoln M, Ferguson A, McAllister L. Issues in developing valid assessments of speech pathology students’ performance in the workplace. Int J Lang Commun Disord. 2010;45: 1–14. 10.3109/13682820902745461 19424886

[78] Judge MP, Polifroni EC, Zhu S. Influence of student attributes on readiness for interprofessional learning across multiple healthcare disciplines: Identifying factors to inform educational development. Int J Nurs Sci. 2015;2: 248–252. 10.1016/j.ijnss.2015.07.007

[79] Yorke M. Formative assessment in higher education: Moves towards theory and the enhancement of pedagogic practice. High Educ. 2003;45: 477–501. 10.1023/A:1023967026413

[80] American Educational Research Association, American Psychological Association, National Council on Measurement in Education. Standards for Educational and Psychological Testing, 2014 Edition American Educational Research Association; 2014.

[81] Carvajal A, Centeno C, Watson R, Martínez M, Rubiales AS. [How is an instrument for measuring health to be validated?]. An Sist Sanit Navar. 2011;34: 63–72. 21532647 10.4321/s1137-66272011000100007

[82] Peeters MJ, Martin BA. Validation of learning assessments: A primer. Curr Pharm Teach Learn. 2017;9: 925–933. 10.1016/j.cptl.2017.06.001 29233326

[83] Skúladóttir H, Svavarsdóttir MH. Development and validation of a Clinical Assessment Tool for Nursing Education (CAT-NE). Nurse Educ Pract. 2016;20: 31–38. 10.1016/j.nepr.2016.06.008 27428801

[84] Grol-Prokopczyk H, Verdes-Tennant E, McEniry M, Ispány M. Promises and Pitfalls of Anchoring Vignettes in Health Survey Research. Demography. 2015;52: 1703–1728. 10.1007/s13524-015-0422-1 26335547 PMC5189702

[85] Knott RJ, Lorgelly PK, Black N, Hollingsworth B. Differential item functioning in quality of life measurement: An analysis using anchoring vignettes. Soc Sci Med. 2017;190: 247–255. 10.1016/j.socscimed.2017.08.033 28881208

[86] Martin E. Vignettes and Respondent Debriefing for Questionnaire Design and Evaluation In: Presser S, Rothgeb JM, Couper MP, Lessler JT, Martin E, Martin J, et al, editors. Wiley Series in Survey Methodology. Hoboken, NJ, USA: John Wiley & Sons, Inc; 2004 pp. 149–171. 10.1002/0471654728.ch8

[87] Dowd JJ. Social Identities Across the Life Course *Social Identities Across the Life Course*, by Hockey Jenny and James Allison. Houndmills, Basingstoke, Hampshire, UK; New York: Palgrave Macmillan, 2003 241 pp. $79.95 cloth. ISBN: 0-333-91283-7. $26.95 paper. ISBN: 0-333-91284-5. Contemp Sociol J Rev. 2004;33: 300–301. 10.1177/009430610403300317

[88] Kelly-Irving M, Soulier A, Mabile L, Bartley M, Raynaud J-P, Panico L, et al Vignettes as tool for research and teaching in life course studies: Interdisciplinary approaches. Adv Life Course Res. 2017;32: 35–41. 10.1016/j.alcr.2016.09.001

[89] Colón-Emeric CS, Corazzini KN, McConnell ES, Pan W, Toles MP, Hall R, et al Resident Vignettes for Assessing Care Quality in Nursing Homes. J Am Med Dir Assoc. 2017; 10.1016/j.jamda.2017.10.018 29174560 PMC5924713

