# Supplementary material for: Development and validation of interprofessional learning assessment tool for health professionals in continuing professional development (CPD)
Source: PLoS One. 2019 Jan 25;14(1):e0211405. doi: 10.1371/journal.pone.0211405 (PMC6347297; doi:10.1371/journal.pone.0211405)
Supplement: S1 Tool — (PDF) [file pone.0211405.s001.pdf]

INVENTORY OF REFLECTIVE VIGNETTE – INTERPROFESSIONAL LEARNING (IRV-IPL)

INSTRUCTION
For each statements below, please rate your answer per column that describes your level of performance as follows:

1 = Emerging; 2 = Developing; 3 = Minimal; 4 = Proficient; 5 = Advanced; 6 = Excellent

IRV-IPL	BEFORE the CPD program, I was able to:						AFTER the CPD program, I am able to:						IF I joined a LECTURE program, I'd be able to:					
**Collaboration**: *purposeful working relationships*	1	2	3	4	5	6	1	2	3	4	5	6	1	2	3	4	5	6
• Work well with the team members																		
• Seek other members to accomplish the work																		
• Include other team members in making plans/decisions																		
• Use a team approach to achieve the goals/outcomes																		
• Explain the roles/tasks of each team member																		
**Coordination**: *faithful mutual performances*	1	2	3	4	5	6	1	2	3	4	5	6	1	2	3	4	5	6
• Negotiate tasks/responsibilities with other participants																		
• Inform other participants for any updates and changes																		
• Work well with the participants of other groups																		
• Discuss your plans/actions with other participants																		
• Know the work/responsibility of other participants																		
**Cooperation**: *helpful personal interactions*	1	2	3	4	5	6	1	2	3	4	5	6	1	2	3	4	5	6
• Share my inputs/abilities with other participants																		
• Be responsible with my contributions to the team																		
• Show my support/concern for other participants																		
• Offer useful information to other participants																		
• Help other participants when necessary																		
**Communication**: *respectful social interactions*	1	2	3	4	5	6	1	2	3	4	5	6	1	2	3	4	5	6
• Listen actively to other participants																		
• Express my concerns in a professional manner																		
• Encourage others to ask useful questions politely																		
• Share my thoughts in a clear effective manner																		
• Manage conflict in a courteous manner																		
**Commendation**: *thoughtful professional appreciations*	1	2	3	4	5	6	1	2	3	4	5	6	1	2	3	4	5	6
• Give constructive feedbacks to other participants																		
• Show trust in other participants while learning/working																		
• Recognize the performance of other participants																		
• Appreciate the contributions of other participants																		
• Consider the inputs/ideas of other participants																		
